# Precision obesity medicine: a translational perspective on epigenetics, the gut microbiome, and AI-assisted multi-omics integration

**DOI:** 10.3389/fgene.2026.1793503

**Published:** 2026-06-23

**Authors:** Ammar A. Jairoun, Sabaa S. Al-Hemyari, Moyad Shahwan, Abeer M. Al-Ghananeem, Araam Al-Salmi, Thantrira Porntaveetus, Amjad Alhalaweh

**Affiliations:** 1 Health and Safety Department, Dubai Municipality, Dubai, United Arab Emirates; 2 Discipline of Clinical Pharmacy, School of Pharmaceutical Sciences, Universiti Sains Malaysia (USM), Pulau Pinang, Malaysia; 3 Pharmacy Department, Emirates Health Services, Dubai, United Arab Emirates; 4 Centre of Medical and Bio-Allied Health Sciences Research, Ajman University, Ajman, United Arab Emirates; 5 Center of Exellence in Precision Medicine and Digital Health, Department of Physiology, Faculty of Dentistry, Chulalongkorn University, Bangkok, Thailand; 6 College of Pharmacy and Health Sciences, Ajman University, Ajman, United Arab Emirates; 7 Dubai Medical University (DMU), College of Medicine, Dubai, United Arab Emirates; 8 Department of Pharmaceutics and Pharmaceutical Technology, College of Pharmacy, University of Sharjah, Sharjah, United Arab Emirates

**Keywords:** AI, epigenetics, gut microbiome, machine learning, obesity medicine, personalized care

## 1 Introduction

As a chronic condition with many moving parts, obesity continues to weigh heavily on global health (World Health Organization, 2024; World Obesity Federation, n.d.). The consequences run well past excess body weight: type 2 diabetes, cardiovascular disease, and several cancers cluster with it (Khaodhiar et al., 1999; Bae et al., 2025). After decades of investigation and drug development, clinicians still struggle to manage the condition consistently, in large part because its onset, course, and response to treatment “differ from one person to the next. Generic “one-size-fits-all” protocols cannot meet that variability, and strategies grounded in each patient’s biology are now seen as necessary.

Momentum has built quickly around precision approaches as multi-omics platforms, microbiome science, and computational modelling have all matured. A bibliometric reading of the literature traces this shift: early genetics-heavy work has given ground to integrative programmes that bring together microbiome profiling, personalized nutrition, multi-omics analysis, and artificial intelligence (AI)-assisted modelling. What is missing, still, is translational glue. Biological profiling, computational modelling, and clinically actionable obesity management have yet to interlock into a coherent framework.

For the purposes of this article, precision obesity medicine is defined as a systems-level clinical practice that links dynamic biological readouts, including epigenetic signatures and functional gut microbiome profiles, to longitudinal clinical, behavioural, environmental, and metabolic data, with AI-driven multi-omics modelling carrying the analytical load. Where conventional stratification leans on static markers such as inherited genetic risk or body mass index (BMI), the framing taken here is different. Reversible regulatory mechanisms, host–microbe interactions, and therapy that adapts as the patient changes sit at the centre.

Against that backdrop, this article weighs the translational prospects of combining epigenetics, gut microbiota composition, and AI-assisted multi-omics analysis for obesity phenotyping and intervention planning. Three threads receive close attention: the mechanistic biology, the methodological constraints, and the work that must come next before any of this can be reliably folded into routine obesity care.

## 2 Bibliometric perspective on precision obesity medicine

To trace how research priorities have shifted, a bibliometric analysis was performed on 282 Scopus-indexed publications spanning obesity, microbiome science, epigenetics, multi-omics integration, personalized nutrition, and AI-assisted modelling. The Supplementary Material carries the full method, including the search strategy, the analytical pipeline, and supporting outputs. Two views of the corpus, keyword co-occurrence and time overlay, show the field expanding rapidly across disciplines, with obesity heterogeneity increasingly tied to computational and molecular profiling work.

**FIGURE 1:**
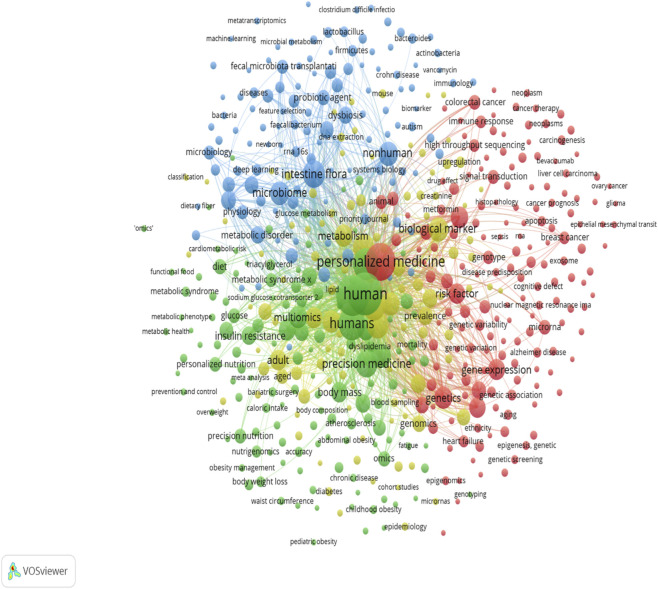
Co-occurrence network of keywords related to precision obesity medicine, microbiome science, epigenetics, personalized nutrition, multi-omics integration, and AI-assisted modelling. Figure 1 displays the keyword co-occurrence network derived from the bibliometric analysis. The network helps clarify how research on precision obesity medicine is currently organized and how far the field has progressed towards translational application. Overall, the pattern suggests that the literature draws on several areas of expertise, although the research remains divided into thematic clusters that are only partly connected.

Within the co-occurrence map, several clusters emerged, linked but not as tightly as one might expect for a single coherent field. The busiest nodes, “precision medicine”, “microbiome”, “gene expression,” “metabolism,” “personalized nutrition,” and “multiomics,” point to a research culture preoccupied with molecular characterisation and the biological stratification of obesity phenotypes. Around the microbiome, three terms “in particular, “dysbiosis,” “intestinal flora,” and “fecal microbiota transplantation,” sat close to clusters dealing with metabolic regulation and personalized nutrition. AI vocabulary tells a different story. Terms such as “machine learning,” “deep learning,” and “multiomics,” sat further out, with thin links to clinical obesity intervention themes. The implication is that AI-driven precision obesity work remains, for the moment, exploratory rather than clinically validated (Figure 1).

**FIGURE 2:**
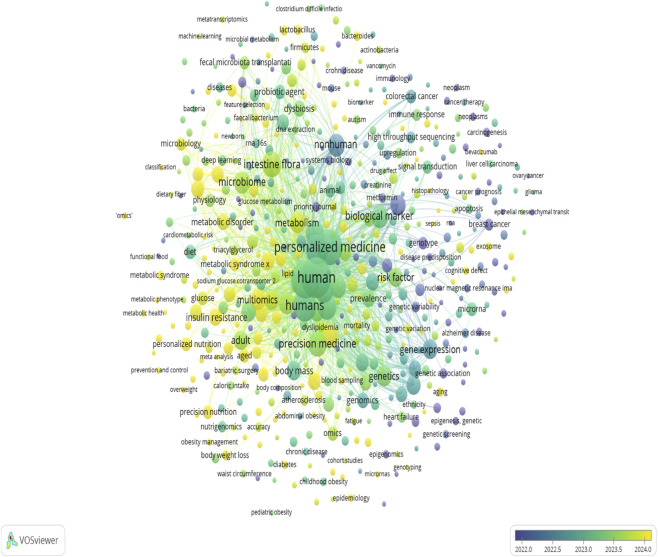
Overlay visualization showing temporal evolution and emerging themes in precision obesity medicine research. Figure 2 shows the overlay visualization of the keyword co-occurrence network and illustrates how research themes in precision obesity medicine developed between 2022 and 2024. The color gradient represents the average publication year of each keyword. Darker blue nodes indicate earlier areas of focus, while yellow nodes represent more recent topics.

Read chronologically, the overlay tells a story of migration. The literature has moved away from earlier work concentrated on genetics and biomarkers, toward newer threads built on microbiome science, personalized nutrition, AI-assisted modelling, and systems-level multi-omics integration. Even so, clinically actionable intervention concepts sat off-centre compared with mechanistic molecular profiling. Taken together, the bibliometric data position precision obesity medicine in the middle of a transition toward integrative translational models, with much still to do on longitudinal validation, reproducibility, interpretability, and decision-support systems clinicians can actually use (Figure 2).

Several terms occupy central positions in the network, including “personalized medicine,” “precision medicine,” “human,” “genetics,” “gene expression,” “microbiome,” “metabolism,” and “multiomics.” Their prominence indicates that much of the current literature is focused on biological classification and molecular description of obesity-related phenotypes. The red cluster contains a high concentration of terms linked to genomics, gene expression, and biomarkers, showing that molecular and genetic profiling remains a major focus of the field.

The blue cluster is organized around microbiome-related terminology, including “microbiome,” “dysbiosis,” “intestinal flora,” “probiotic agent,” and “fecal microbiota transplantation.” This grouping reflects the growing research interest in host-microbial relationships and their influence on metabolic regulation. The green cluster is more closely associated with metabolic and clinical concepts, including “insulin resistance,” “metabolic syndrome,” “body mass,” “personalized nutrition,” and “obesity management.” This indicates increasing attention to intervention strategies that are more responsive to clinical and nutritional differences between individuals.

Although “machine learning,” “deep learning,” and “multiomics” appear in the network, these terms are positioned more towards the margins and show weaker connections with the main biological clusters. This supports the Editor-in-Chief’s concern that AI-based precision obesity models are still in an early stage of development and have not yet been fully incorporated into the wider research structure.

The distance between the molecular, microbiome, and clinical-management clusters also suggests that the field has not yet established a fully integrated translational framework. In particular, there is still limited connection between longitudinal biological data, behavioral information, treatment responses, and clinically useful decision-making tools. The bibliometric findings therefore support the main argument of the revised manuscript: while epigenetics, microbiome research, and AI are all expanding within obesity medicine, their practical integration remains limited. Further methodological consistency, long-term validation, and clinically transparent analytical models are needed before precision obesity medicine can be applied as part of routine clinical care.

The visualization shows a movement away from research centered mainly on genetics and biomarkers towards broader approaches that include microbiome science, multi-omics analysis, and personalized nutrition. Earlier work was concentrated around keywords such as “gene expression,” “genetic screening,” “genotype,” “epigenesis,” and “genetic association.” This pattern indicates that the field initially gave considerable attention to molecular description and genetic susceptibility.

More recent keywords, including “multiomics,” “personalized nutrition,” “microbiome,” “deep learning,” “machine learning,” “diet,” “metabolic syndrome,” and “precision medicine,” appear in later phases of the network. This suggests that the field is beginning to move towards more integrated models that combine biological, metabolic, dietary, and computational data.

Despite this shift, AI-related and microbiome-related terms remain relatively dispersed across the network. Their limited connection with other areas suggests that the combination of computational modelling, microbiome profiling, and biologically informed obesity management is still developing and has not yet become a coherent research framework.

The overlay visualization also shows that concepts linked to direct clinical application are less central than those associated with molecular profiling. This indicates that current research remains more focused on mechanisms and exploratory analysis than on implementation in everyday clinical settings.

These findings support the revised direction of the manuscript by showing that precision obesity medicine is moving from separate molecular and genetic studies towards more integrated multi-omics and AI-supported approaches. At the same time, the analysis identifies continuing gaps in long-term validation, translational integration, and clinically interpretable decision-support tools. The bibliometric evidence therefore points to the need for standardized datasets, collaboration across disciplines, and carefully validated translational models before precision obesity medicine can be used routinely in obesity care.

## 3 Biological and translational foundations of precision obesity medicine

### 3.1 Epigenetic and microbiome interactions

Epigenetics sits at the join between inherited susceptibility and lived exposure. Without rewriting the DNA itself, mechanisms such as DNA methylation, histone modifications, and non-coding RNA regulation steer adipogenesis, inflammation, insulin sensitivity, and metabolic adaptation (Nohesara et al., 2025; Mahmoud, 2022; King and Skinner, 2020; Long et al., 2024; Gao et al., 2021; Jung and Kang, 2021; Klimczak and Śliwińska, 2024; Samblas et al., 2019; Ren et al., 2024; Hinte et al., 2024; Hinte et al., 2025). Several recent lines of evidence go further, suggesting that a kind of epigenetic “memory” lingers and may help explain why weight is so often regained after a successful loss.

The gut microbiome contributes to host metabolism on several fronts: digesting nutrients, regulating immune activity, processing bile acids, and producing bioactive compounds such as short-chain fatty acids (SCFAs) (Zhang et al., 2015; Rinninella et al., 2019; Turnbaugh et al., 2006; Ley et al., 2006; Li et al., 2020; Karlsson et al., 2013; Cani et al., 2008; Henao-Mejia et al., 2012; Vrieze et al., 2012; Dao et al., 2016; Morais et al., 2021; Sharma et al., 2025). When obesity is accompanied by dysbiosis, the consequences may include a compromised intestinal barrier, low-grade systemic inflammation, and altered energy extraction and metabolic signalling. There is, intriguingly, a tighter link. Microbial metabolites can act directly on host epigenetic machinery, pointing to a mechanistic thread that runs from microbiome function to the heterogeneity of obesity itself.

The bibliometric data echo this convergence: microbiome science, personalized nutrition, and multi-omics integration are gravitating toward each other. Both epigenetic and microbiome research, even so, sit closer to mechanistic enquiry than to clinically validated obesity intervention frameworks.

### 3.2 AI-assisted multi-omics integration

Datasets produced by genomics, epigenomics, metabolomics, proteomics, and metagenomics are large, layered, and difficult to handle with classical statistics; artificial intelligence and machine learning offer a way to read them at scale. In exploratory studies of obesity phenotypes and variable treatment responses, supervised techniques have already shown promise. Random forests, elastic net regression, gradient boosting algorithms, and deep neural networks have all surfaced in this work.

The methodological terrain, however, is uneven. Multi-omics studies routinely deal with many more features than participants, which inflates the risk of overfitting and blunts reproducibility once a model meets a new population (Saikia and Kalita, 2025). Microbiome prediction models inherit additional sources of noise. Sequencing methods, bioinformatic processing, environmental exposure, and demographic diversity all leave their mark. Newer techniques such as Wasserstein Generative Adversarial Networks with Gradient Penalty (WGAN-GP) hold some promise for generalisation (Wang and Chang, 2025), but robust external validation and longitudinal reproducibility are not yet in place.

Interpretability is the other obstacle to clinical use. Tools for explainable AI, including SHAP and LIME, can lift the lid on which features matter for a given prediction and so, in principle, help clinicians decide and trust (Hasan et al., 2024; Ghalandari et al., 2025). The bibliometric data temper this picture. AI research and clinically actionable obesity intervention frameworks remain only partially joined, which keeps current applications largely in the realm of the exploratory.

### 3.3 Translational opportunities and limitations

Joining epigenetics, microbiome science, and AI-assisted multi-omics analysis may help identify obesity phenotypes that are biologically informed and capture differences in how individuals respond to treatment. With that information in hand, adaptive interventions become more plausible. Choices about dietary composition, meal timing, fibre subtype selection, microbiome-directed therapies, behavioural change, and pharmacotherapy could all be guided more precisely.

The evidence base, though, is bounded by methodological heterogeneity, cohort-specific quirks, sparse external validation, and short follow-up. Integrative multi-omics approaches therefore open genuine opportunities for refining obesity phenotyping and individualized intervention planning, but clinical adoption hinges on pieces that are not yet in place: harmonised datasets, reproducible biomarkers, interpretable analytical systems, and prospective studies designed to a high standard.

## 4 Methodological and ethical challenges

Precision obesity medicine is still early in its translational arc, and the road forward is crowded with methodological, clinical, and ethical obstacles. Findings from multi-omics studies are sensitive to technical detail. Platform differences, batch effects, population stratification, and variability in tissue sampling and microbiome sequencing all bend the results (Carter and Sobey, 2025). Many of the biomarkers themselves are moving targets. Epigenetic and microbial readouts shift with diet, stress, medication, and metabolic state (Chanda and De, 2024; Chatterjee et al., 2021), which is why longitudinal validation matters as much as it does.

To date, most AI-based obesity prediction models have been validated only on the data they were trained on; reproducibility across independent populations and clinical settings has been thin (Chatterjee et al., 2021). Bridging from algorithm to practice will demand external validation, prospective interventional studies, economic evaluation, and regulatory scrutiny. Ethical questions sit alongside the technical ones. Informed consent, data privacy, algorithmic bias, and inequitable access to advanced technologies all need careful handling (Piening et al., 2018; Savla et al., 2025). None of this can replace public health work on the environmental, socioeconomic, behavioural, and commercial determinants of obesity; precision approaches should add to those broader strategies, not substitute for them (Wang, 2026; Chatelan et al., 2019).

The Supplementary Material houses the full bibliometric methodology: the Scopus search query, the VOSviewer analytical procedures, keyword thresholds, validation processes, and the data extraction workflow.

## 5 Conclusion

Precision obesity medicine is a translational project in progress, aimed at sharpening how obesity is classified and how individual interventions are planned by drawing epigenetic science, gut microbiome research, and AI-assisted multi-omics analysis into a single picture. The bibliometric record shows the science converging around microbiome function, personalized nutrition, computational modelling, and systems-level biological profiling. The same record confirms that the field is not yet mature. Exploratory molecular research and clinically actionable obesity management frameworks still sit on either side of a gap that has yet to be closed.

Biological relevance is no longer in serious doubt. Epigenetic regulation and host–microbe interactions feature credibly in obesity heterogeneity and in how patients respond to treatment. What remains is the harder work: methodological heterogeneity, reproducibility, external validation, interpretability, and ethical implementation. Real progress will turn on four pieces falling into place: harmonised longitudinal cohorts, clinically actionable biomarker strategies, interpretable AI frameworks, and intervention studies of sufficient rigour to show measurable gains over today’s standard of care.
