# Supplementary material for: Precision obesity medicine: a translational perspective on epigenetics, the gut microbiome, and AI-assisted multi-omics integration
Source: Front Genet. 2026 Jun 23;17:1793503. doi: 10.3389/fgene.2026.1793503 (PMC13372542; doi:10.3389/fgene.2026.1793503)
Supplement: Supplementary file 1 [file DataSheet1.pdf]

The advanced search functionality in Scopus was employed with the following query:

TITLE-ABS-KEY ((obes* OR "weight gain" OR adiposity OR overweight) AND ("precision medicine" OR "personalized medicine" OR "personalised medicine" OR "precision nutrition" OR "personalized nutrition") AND (epigenetic* OR methylation OR "DNA methylation" OR histone* OR "non-coding RNA" OR miRNA OR microbiome OR microbiota OR metagenom* OR metabolom* OR proteom*) AND ("artificial intelligence" OR "machine learning" OR "deep learning" OR "neural network*" OR "multi-omics" OR "omics integration" OR bioinformatic*)) AND (EXCLUDE (DOCTYPE , "le") OR EXCLUDE (DOCTYPE , "no") OR EXCLUDE (DOCTYPE , "ed"))

The initial search focused on articles containing relevant terms in their title, abstract, or keywords, identifying 300 publications from 2010 to 2026. After applying inclusion criteria—which restricted the selection to peer-reviewed, English-language journal articles specifically addressing microbiome-related metabolic regulation, multi-omics integration, personalized nutrition, and AI-assisted analytical modelling —withdrawn and errata manuscripts were excluded. The final dataset consisted of 282 articles.

To minimize bias from database updates, data extraction was finalized on May11, 2026. These studies were then analyzed to identify evolving research trends over time, examine co-occurrence networks of keywords related to microbiome-related metabolic regulation, multi-omics integration, personalized nutrition, and AI-assisted analytical modelling.

Titles, abstracts, and full texts were manually reviewed to ensure relevance and exclude extraneous articles. To validate the search query, two bibliometric specialists analyzed the five most-cited papers, confirming the absence of false positives. A correlation test comparing the retrieved dataset with actual findings from the 5 most active researchers in the field demonstrated a strong correlation ($r = 0.812$, $p < 0.001$), further affirming the validity and accuracy of the search strategy.

The curated dataset was exported in CSV format for analysis using Microsoft Excel. It included details on titles, abstracts, authorship, institutional affiliations, publication years, document types, funding agencies, citations, and journal names. VOSviewer software (Version 1.6.20) was then employed to generate network maps

SHARED – CONFIDENTIAL / خاص – مشترك

illustrating term co-occurrence in titles and abstracts, along with collaboration patterns among countries. Co-occurrence analysis grouped terms into distinct clusters, each marked with unique colors, facilitating the identification of research hotspots and emerging trends. This approach provided a comprehensive understanding of the bibliometric landscape and enabled the prediction of future research trajectories.

Visualization of Co-occurrence network

Figure 1 displays the keyword co-occurrence network derived from the bibliometric analysis. The network helps clarify how research on precision obesity medicine is currently organized and how far the field has progressed towards translational application. Overall, the pattern suggests that the literature draws on several areas of expertise, although the research remains divided into thematic clusters that are only partly connected.

Several terms occupy central positions in the network, including “personalized medicine,” “precision medicine,” “human,” “genetics,” “gene expression,” “microbiome,” “metabolism,” and “multiomics.” Their prominence indicates that much of the current literature is focused on biological classification and molecular description of obesity-related phenotypes. The red cluster contains a high concentration of terms linked to genomics, gene expression, and biomarkers, showing that molecular and genetic profiling remains a major focus of the field.

The blue cluster is organized around microbiome-related terminology, including “microbiome,” “dysbiosis,” “intestinal flora,” “probiotic agent,” and “fecal microbiota transplantation.” This grouping reflects the growing research interest in host-microbial relationships and their influence on metabolic regulation. The green cluster is more closely associated with metabolic and clinical concepts, including “insulin resistance,” “metabolic syndrome,” “body mass,” “personalized nutrition,” and “obesity management.” This indicates increasing attention to intervention strategies that are more responsive to clinical and nutritional differences between individuals.

Although “machine learning,” “deep learning,” and “multiomics” appear in the network, these terms are positioned more towards the margins and show weaker connections with the main biological clusters. This supports the Editor-in-Chief’s concern that AI-based precision obesity models are still in an early stage of development and have not yet been fully incorporated into the wider research structure.

SHARED – CONFIDENTIAL / خاص – مشترك

The distance between the molecular, microbiome, and clinical-management clusters also suggests that the field has not yet established a fully integrated translational framework. In particular, there is still limited connection between longitudinal biological data, behavioral information, treatment responses, and clinically useful decision-making tools. The bibliometric findings therefore support the main argument of the revised manuscript: while epigenetics, microbiome research, and AI are all expanding within obesity medicine, their practical integration remains limited. Further methodological consistency, long-term validation, and clinically transparent analytical models are needed before precision obesity medicine can be applied as part of routine clinical care.

SHARED – CONFIDENTIAL / خاص – مشترك

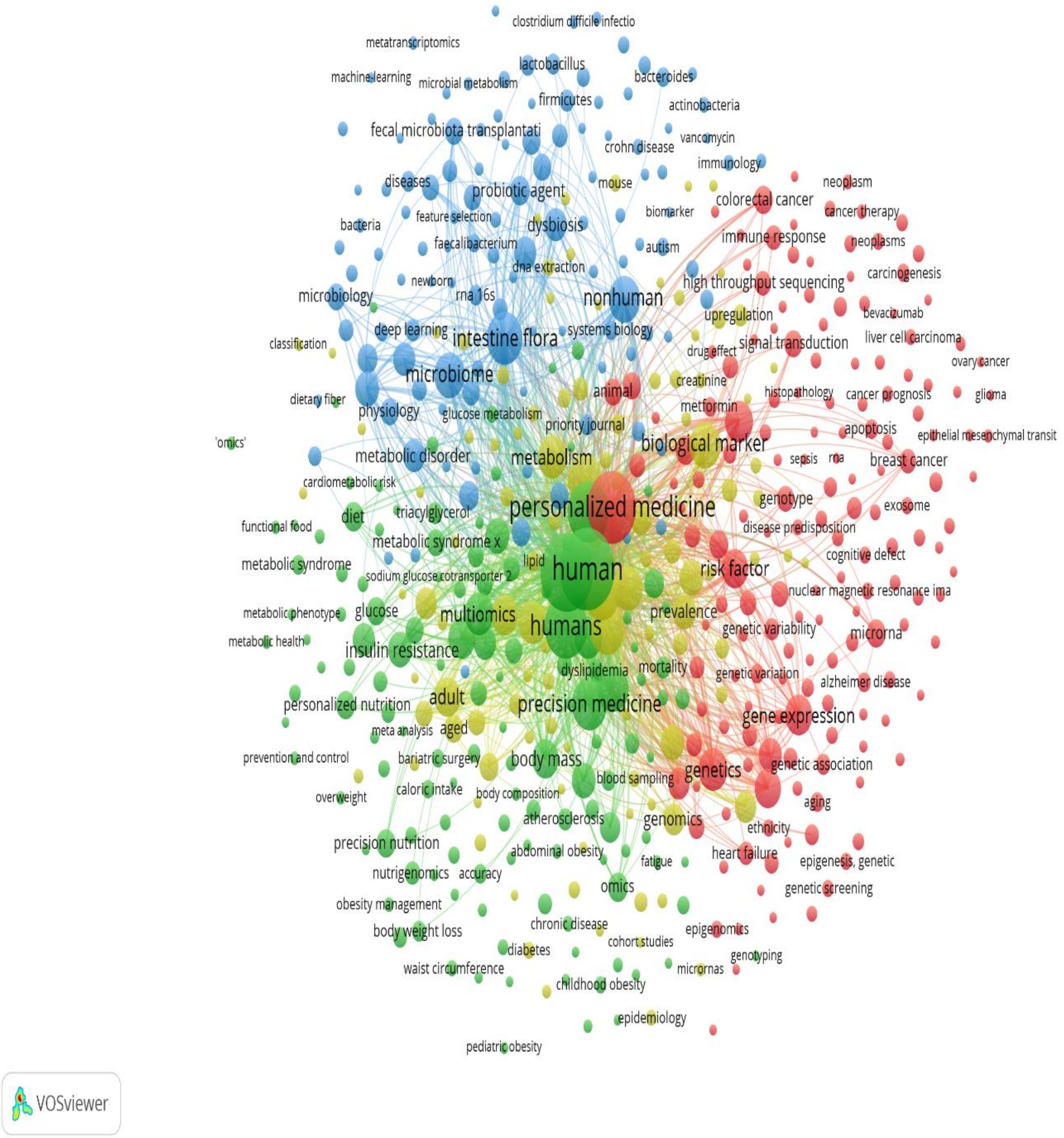

SHARED – CONFIDENTIAL / مشترك – خاص

Overlay Visualization and Emerging Research Trends

Figure 2 shows the overlay visualization of the keyword co-occurrence network and illustrates how research themes in precision obesity medicine developed between 2022 and 2024. The color gradient represents the average publication year of each keyword. Darker blue nodes indicate earlier areas of focus, while yellow nodes represent more recent topics.

The visualization shows a movement away from research centered mainly on genetics and biomarkers towards broader approaches that include microbiome science, multi-omics analysis, and personalized nutrition. Earlier work was concentrated around keywords such as “gene expression,” “genetic screening,” “genotype,” “epigenesis,” and “genetic association.” This pattern indicates that the field initially gave considerable attention to molecular description and genetic susceptibility.

More recent keywords, including “multiomics,” “personalized nutrition,” “microbiome,” “deep learning,” “machine learning,” “diet,” “metabolic syndrome,” and “precision medicine,” appear in later phases of the network. This suggests that the field is beginning to move towards more integrated models that combine biological, metabolic, dietary, and computational data.

Despite this shift, AI-related and microbiome-related terms remain relatively dispersed across the network. Their limited connection with other areas suggests that the combination of computational modelling, microbiome profiling, and biologically informed obesity management is still developing and has not yet become a coherent research framework.

The overlay visualization also shows that concepts linked to direct clinical application are less central than those associated with molecular profiling. This indicates that current research remains more focused on mechanisms and exploratory analysis than on implementation in everyday clinical settings.

These findings support the revised direction of the manuscript by showing that precision obesity medicine is moving from separate molecular and genetic studies towards more integrated multi-omics and AI-supported approaches. At the same time, the analysis identifies continuing gaps in long-term validation, translational integration, and clinically interpretable decision-support tools. The bibliometric evidence therefore points to the need for standardized datasets, collaboration across disciplines, and carefully validated translational models before precision obesity medicine can be used routinely in obesity care.

SHARED – CONFIDENTIAL / خاص – مشترك

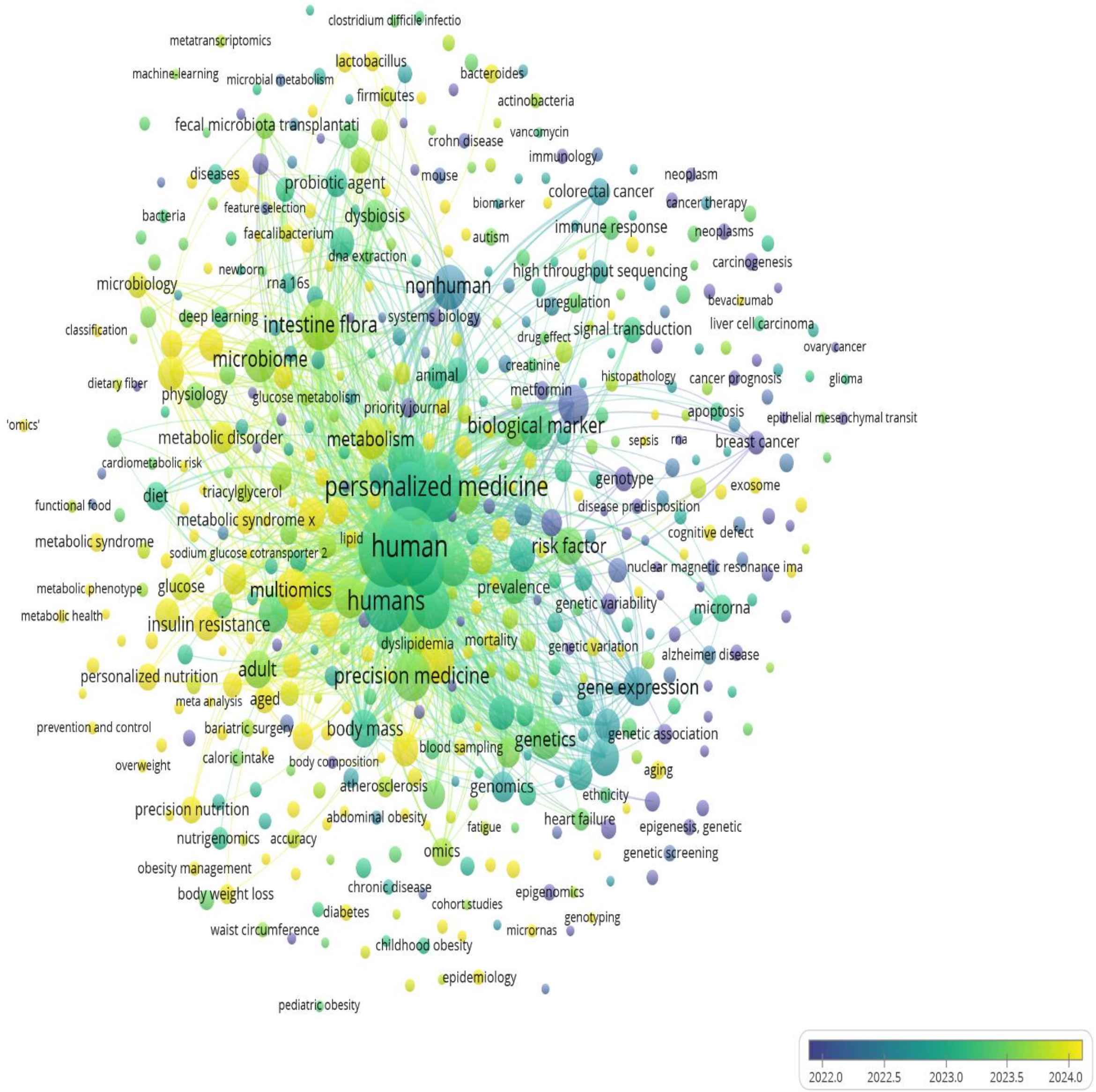
clostridium difficile infectio
metatranscriptomics
machine learning
microbial metabolism
lactobacillus
bacteroides
firmicutes
actinobacteria
fecal microbiota transplantati
vancomycin
crohn disease
immunology
diseases
probiotic agent
mouse
neoplasm
colorectal cancer
feature selection
biomarker
cancer therapy
bacteria
dysbiosis
faecalibacterium
autism
immune response
neoplasms
dna extraction
carcinogenesis
newborn
high throughput sequencing
microbiology
rna 16s
nonhuman
upregulation
bevacizumab
deep learning
systems biology
liver cell carcinoma
classification
intestine flora
drug effect
signal transduction
ovary cancer
microbiome
creatinine
dietary fiber
animal
histopathology
cancer prognosis
glioma
physiology
glucose metabolism
metformin
priority journal
apoptosis
'omics'
epithelial mesenchymal transit
biological marker
metabolic disorder
metabolism
sepsis
rna
breast cancer
cardiometabolic risk
genotype
exosome
personalized medicine
diet
triacylglycerol
functional food
disease predisposition
metabolic syndrome x
cognitive defect
metabolic syndrome
lipid
human
risk factor
sodium glucose cotransporter 2
nuclear magnetic resonance ima
metabolic phenotype
glucose
multiomics
prevalence
humans
genetic variability
microrna
metabolic health
insulin resistance
dyslipidemia
mortality
genetic variation
alzheimer disease
adult
personalized nutrition
precision medicine
gene expression
meta analysis
aged
prevention and control
bariatric surgery
body mass
genetic association
genetics
blood sampling
caloric intake
overweight
body composition
aging
atherosclerosis
genomics
ethnicity
precision nutrition
abdominal obesity
heart failure
epigenesis, genetic
nutrigenomics
accuracy
fatigue
omics
genetic screening
obesity management
body weight loss
chronic disease
epigenomics
cohort studies
diabetes
genotyping
waist circumference
micrornas
childhood obesity
epidemiology
pediatric obesity
VOSviewer
2022.0
2022.5
2023.0
2023.5
2024.0
